# Functional inhibition of NF-κB signal transduction in αvβ3 integrin expressing endothelial cells by using RGD-PEG-modified adenovirus with a mutant IκB gene

**DOI:** 10.1186/ar1885

**Published:** 2006-01-13

**Authors:** Ken-ichi Ogawara, Joanna M Kułdo, Koen Oosterhuis, Bart-Jan Kroesen, Marianne G Rots, Christian Trautwein, Toshikiro Kimura, Hidde J Haisma, Grietje Molema

**Affiliations:** 1 Department of Pharmaceutics, Faculty of Pharmaceutical Sciences, Okayama University, Okayama, Japan; 2 University of Groningen, Department of Pathology and Laboratory Medicine, Medical Biology Section, The Netherlands; 3 Department of Therapeutic Gene Modulation, Groningen University Institute for Drug Exploration, Groningen, The Netherlands; 4 III Medical Clinic, University Hospital of RWTH, Aachen, Germany

## Abstract

In order to selectively block nuclear factor κB (NF-κB)-dependent signal transduction in angiogenic endothelial cells, we constructed an αvβ3 integrin specific adenovirus encoding dominant negative IκB (dnIκB) as a therapeutic gene. By virtue of RGD modification of the PEGylated virus, the specificity of the cell entry pathway of adenovirus shifted from coxsacki-adenovirus receptor dependent to αvβ3 integrin dependent entry. The therapeutic outcome of delivery of the transgene into endothelial cells was determined by analysis of cellular responsiveness to tumor necrosis factor (TNF)-α. Using real time reverse transcription PCR, mRNA levels of the cell adhesion molecules E-selectin, vascular cell adhesion molecule (VCAM)-1 and intercellular adhesion molecule (ICAM)-1, the cytokines/growth factors IL-6, IL-8 and vascular endothelial growth factor (VEGF)-A, and the receptor tyrosine kinase Tie-2 were assessed. Furthermore, levels of ICAM-1 protein were determined by flow cytometric analysis. RGD-targeted adenovirus delivered the dnIκB via αvβ3 to become functionally expressed, leading to complete abolishment of TNF-α-induced up-regulation of E-selectin, ICAM-1, VCAM-1, IL-6, IL-8, VEGF-A and Tie-2. The approach of targeted delivery of dnIκB into endothelial cells presented here can be employed for diseases such as rheumatoid arthritis and inflammatory bowel disease where activation of NF-κB activity should be locally restored to basal levels in the endothelium.

## Introduction

Microvascular endothelial cells are active participants in a variety of diseases, including cancer [1] and chronic inflammation such as rheumatoid arthritis [2]. In inflammatory reactions, endothelial cells facilitate transmigration of leukocytes by expression of cell adhesion molecules such as E-selectin, vascular cell adhesion molecule (VCAM-1) and intercellular adhesion molecule (ICAM-1), as well as production of cytokines and chemokines [3]. Inflammatory mediators can also, either directly or indirectly, promote angiogenesis. Moreover, several observations suggest that angiogenesis and inflammation proceed in a co-ordinated fashion and sustain one another during chronic inflammatory diseases and in cancer growth [4]. Thus, their active roles in the pathophysiology of disease, together with their easy accessibility in the blood, makes endothelial cells attractive target cells for therapy.

Nuclear factor κB (NF-κB)/Rel transcription factors represent a ubiquitously expressed protein family that modulates the expression of genes involved in diverse cellular functions, such as stress response, innate and adaptive immune reactions, and apoptosis [5-8]. In endothelial cells, NF-κB is activated by inflammatory cytokines, bacterial lipopolysaccharides, oxidized low-density lipoprotein, advanced glycation end products, platelet-derived growth factor, and hypoxia/reoxygenation, among others. Rheumatoid arthritis, inflammatory bowel disease and other chronic inflammatory processes have been associated with elevated levels of endothelial NF-κB [9-13].

A dominant negative form of IκB (dnIκB) that contains serine-to-alanine mutations at amino acids 32 and 36 blocks endogenous IκB phosphorylation and subsequent proteosome-mediated degradation, thereby inhibiting NF-κB mediated gene expression [14]. To achieve selective gene transfer of dnIκB into endothelial cells, adenovirus can be used as a vector. Infection by adenovirus is initiated by the high affinity binding of the carboxy-terminal 'knob' part of the fiber protein to coxsacki-adenovirus receptor (CAR), thereby limiting its infection specificity to CAR-positive cells. In a previous study, we showed that PEGylation of the adenovirus and subsequent conjugation with anti-E-selectin antibody as a homing ligand coupled onto the distal functional group of polyethylene glycol (PEG) could selectively deliver a reporter gene into activated endothelial cells *in vivo*. The modulated virus-target cell interaction took place via recognition of E-selectin on activated endothelium by the homing ligand, thereby evading the endogenous CAR-based tropism of the virus [15]. In the present study, we constructed an RGD-modified, αvβ3 integrin specific adenovirus encoding dnIκB as a therapeutic gene to block NF-κB-dependent signal transduction in endothelial cells. Integrin specificity of RGD-modified adenovirus with respect to its gene transfer and transgene expression was evaluated by western blot analysis. Pharmacological effectiveness of delivery and expression of the transgene into endothelial cells was studied using real time reverse transcription (RT)-PCR and flow cytometric analysis of pro-inflammatory and pro-angiogenic gene expression profiles in tumor necrosis factor (TNF)-α activated endothelial cells.

## Materials and methods

### Chemicals and proteins

#### RGD and control peptides

The cyclic RGD-peptide c(RGDf(∊*-S*-acetylthioacetyl)K) and the RAD analogue c(RADf(∊*-S*-acetylthioacetyl)K), hereafter referred to as RGDpep and RADpep, respectively, were prepared by Ansynth (Roosendaal, The Netherlands). This RGDpep was previously conjugated to a humanized antibody that does not recognize any epitope relevant for the cells under study (hereafter referred to as RGD-protein). RGD conjugation provided the protein with αvβ3 integrin specificity [16].

#### Production of knob5

The knob domains of adenovirus5 fibers were expressed in *Escherichia coli *with amino-terminal His6 tags, using the pQE30 expression vector (Qiagen, Hilden, Germany) [17]. Knob5 was purified on Ni-nitrilotriacetic acid agarose columns (Qiagen) and dialyzed against PBS. The ability of knob5 to form homotrimers was verified by SDS-PAGE of boiled and unboiled samples. The concentration of the purified knob5 was determined by the Bradford protein assay (Bio-Rad, Hercules, CA, USA) using bovine serum albumin as the standard.

### Cells

#### Endothelial cells

Human umbilical vein endothelial cells (HUVECs) were obtained from the Endothelial Cell Facility UMCG (Groningen, The Netherlands). Primary isolates were cultured on 1% gelatin-precoated tissue culture flasks (Costar, The Netherlands) at 37°C under 5% CO_2_/95% air. The endothelial cell culture medium consisted of RPMI 1640 supplemented with 20% heat inactivated FCS, 2 mM L-glutamine, 5 U/ml heparin, 100 U/ml penicillin, 100 μg/ml streptomycin, and 50 μg/ml endothelial cell growth factor supplement extracted from bovine brain. Upon confluence, cells were detached from the surface by trypsin/EDTA (0.5/0.2 mg/ml in PBS; GibcoTM, Paisley, Scotland, UK) and split at a 1:3 ratio. For the experiments described, HUVECs were used up to passage four.

### Viruses

The recombinant replication-deficient adenovirus encoding dominant negative form of IκB under control of the cytomegalovirus (CMV) promoter, hereafter referred to as AddnIκB, contains a hemagglutinin (HA)-tagged super-repressor IκB. This super-repressor IκB has serine-to-alanine mutations in residues 32 and 36, which inhibit its phosphorylation and proteosome-mediated degradation [14]. Virus was grown on HEK293 cells and purified in Hepes/sucrose buffer, pH 8.0, according to conventional double CsCl gradient centrifugation methods, and the number of viral particles was calculated from the optical density at 260 nm (OD_260_). AdLacZ, which contains the *E. coli *β-galactosidase gene, was grown and purified as described above and used as a control virus. Standard plaque assays were performed to determine the viral particles (vp)/plaque forming unit ratio, which were found to be 15 for both viruses.

#### Chemical conjugation of adenovirus

Conjugation reactions were performed as reported previously [15]. In brief, an aliquot of heterobifunctional polyethylene glycol (PEG) linker (3.4 kDa) with a N-hydroxysuccinimide ester and vinyl sulfone group at each end of the molecule (NEKTAR Therapeutics, Huntsville, AL, USA) dissolved in dimethyl formamide (DMF) (100 mg/1 ml DMF) was added slowly to the virus (1 × 10^12 ^viral particles) in a ratio of 10^5^:1 moles PEG:viral particles. The reaction mixture was protected from light and gently mixed for 1.5 hours at 4°C. After the purification using a PD-10 column (Amersham Biotech, Uppsala, Sweden), PEGylated virus was directly used in the following coupling reaction with either RGDpep or RADpep. RGDpep or RADpep dissolved in an acetonitrile-water mixture (1:4) at a concentration of 10 mg/ml was added dropwise to the PEGylated virus in the molar ratio of 10^5^:1. After the addition of 25 μl of a freshly prepared 1 M hydroxylamine solution to deprotect the thiol group of the peptide, the mixture was reacted for four hours at 4°C under gentle mixing. Unreacted reagents were removed by dialysis (DispoDialyzers 300 KD MWCO, Spectrum Laboratories, Rancho Dominguez, CA, USA) against Hepes/sucrose buffer (pH 8.0) at 4°C. Initial studies showed that in the PD-10 column purification procedure, the first 80% of the peak containing PEGylated virus that eluted from the column was free from contamination with unconjugated PEG, and that the dialysis procedure did not lead to loss of conjugated virus. Therefore, we collected the initial 80% of PEGylated virus that eluted from the PD-10 column and used the factor of 0.8 to calculate the final number of viral particles of each preparation. The final virus preparation was collected and stored at -80°C in small aliquots until use.

### Transduction protocol

For the transduction experiments, HUVECs were plated at 12,500 cells/cm^2 ^in 25 cm^2^-tissue culture flasks (Costar, Cambridge, MA, USA) for western blotting, or in 6-well tissue culture plates (Costar) for flow cytometric analysis and real time RT-PCR, and cultured overnight before starting the experiments. The various viral vectors diluted in Dulbecco's modified Eagle's medium without FCS were added to the HUVECs and incubated for 90 minutes at 37°C. The medium was then replaced by normal endothelial cell culture medium and cells were incubated for another 24 hours to allow transgene production. In the case of competition experiments, cells were incubated with RGD-protein (50 μg/ml), recombinant knob5 (20 μg/ml), or both for 30 minutes at 4°C prior to the addition of viruses.

### Western blot analysis of dnIκB in HUVECs

HUVECs were infected with AddnIκB, AddnIκB-PEG-RGD or AddnIκB-PEG-RAD (3,000 vp/cell) as described. After another 24 hours of culturing, cells were detached from the surface by trypsin/EDTA treatment, lysed in cell culture lysis reagent (Promega Corporation, Madison, WI, USA) and sonicated twice for five seconds. After centrifugation for ten minutes at 10,000g, cleared cell lysates were collected and protein content was determined using the Bradford protein assay reagent (Bio-Rad Laboratories, Hercules, CA, USA), using bovine serum albumin as the standard. Samples were then mixed 1:1 with 2 × SDS sample buffer, boiled for 5 minutes, and 30 μg was loaded on SDS-PAGE 10% acrylamide gels followed by blotting to nitrocellulose membranes (Bio-Rad Laboratories). The dnIκB protein was detected using a rabbit anti-HA-tag antibody (sc805; Santa Cruz Biotechnology, Santa Cruz, CA, USA), while both endogenous IκB and dnIκB were detected using a rabbit anti-IκB antibody (sc847; Santa Cruz Biotechnology). Blots were blocked in blocking buffer (5% non-fat drymilk in PBS) for two hours, incubated for one hour with primary antibody diluted in blocking buffer 1:200 (sc805) or 1:250 (sc847) and subsequently with horseradish peroxidase-conjugated swine anti-rabbit antibody (Dako, Glostrup, Denmark) diluted in blocking buffer 1:2,000. Detection was performed using ECL detection reagents (Amersham Corp., Arlington Heigths, IL, USA) according to the manufacturer's protocol.

### RNA isolation and real time RT-PCR analysis

HUVECs were infected with AddnIκB and AddnIκB-PEG-RGD at 7,500 vp/cell as described. After another 24 hours of culturing, cells were activated with 100 ng/ml TNF-α (Boehringer, Ingelheim, Germany), or left resting. Total RNA was isolated 24hours after activation using the Absolutely RNA Microprep Kit (Stratagene, Amsterdam, The Netherlands) according to the protocol of the manufacturer. RNA yield (OD_260_) and purity (OD_260_/_280_) was measured using a ND-1000 UV- Vis Spectrophotometer (NanoDrop Technologies, Rockland, DE, USA). One μg total cellular RNA was subsequently used for the synthesis of first strand cDNA using SuperScript III RNase H^- ^Reverse Transcriptase (Invitrogen, Breda, The Netherlands) in 20 μl final volume containing 250 ng random hexamers (Promega) and 40 units RNase OUT inhibitor (Invitrogen). After RT-reaction, cDNA was diluted with distilled water to 100 μl. The following exons overlapping primers and minor groove binder (MGB) probes used for real time RT-PCR were purchased as Assay-on-Demand from Applied Biosystems (Nieuwekerk a/d IJssel, The Netherlands): housekeeping gene GAPDH (assay ID Hs99999905_m1), endothelial cell marker CD31 (PECAM-1 (platelet endothelial cell adhesion molecule 1), Hs00169777_m1), E-selectin (Hs00174057_m1), VCAM-1 (Hs00365486_m1), ICAM-1 (Hs00164932_m1), IL-6 (Hs00174131_m1), IL-8 (Hs00174103_m1), Hs00173626_m1 (hVEGF-A), and Hs00176096 (hTie-2). The final concentration of primers and MGB probes in TaqMan PCR MasterMix (Applied Biosystems, Foster City, CA, USA) for each gene was 900 nM and 250 nM, respectively. As a control, RNA samples not subjected to reverse transcriptase were analyzed to exclude unspecific signals arising from genomic DNA. Those samples consistently showed no amplification signals.

TaqMan real time RT-PCR was performed in an ABI PRISM 7900HT Sequence Detector (Applied Biosystems). Amplification was performed using the following cycling conditions: 2 minutes at 50°C, 10 minutes at 95°C, and 40 to 45 two-step cycles of 15 seconds at 95°C and 60 s at 60°C. Triplicate real time RT-PCR analyses were executed for each sample, and the obtained threshold cycle (Ct) values were averaged. According to the comparative Ct method described in the ABI manual, gene expression was normalized to the expression of the housekeeping gene GAPDH, yielding the ΔCt value. The average ΔCt value obtained from resting HUVECs was then subtracted from the average ΔCt value of each corresponding sample subjected to TNF-α stimulation, yielding the ΔΔCt value. The gene expression level, normalized to the housekeeping gene, and relative to the control sample, was calculated by 2^-ΔΔCt^. Data were normalized to untreated, non-activated control HUVECs arbitrarily set at 1.

In our preliminary analysis, we used CD31 as a housekeeping gene since it is constitutively expressed in HUVECs and its expression is NF-κB-independent (JM Kuldo and G Molema, unpublished data. The outcome for all genes studied remained the same as when GAPDH was used as the housekeeping gene. We therefore regarded GAPDH as a good housekeeping gene for use in the experimental conditions used in this study.

### Flow cytometric analysis of ICAM expression

HUVECs were infected with AddnIκB (7,500 vp/cell), AdLacZ (7,500 vp/cell) and AddnIκB-PEG-RGD at different amounts of vp/cell as described. After another 24 hours of culturing, cells were activated with 100 ng/ml TNF-α (Boehringer) or left resting. Cells were detached from the surface by trypsin/EDTA 4 hours after activation and resuspended in PBS with 5% FCS. Cells were subsequently centrifuged at 200 × g, after which the cell pellets were incubated for 1 hour at 37°C with 100 μl of primary antibody. The following primary antibodies were used: mouse anti-human ICAM (5/3-2.1, kindly provided by Dr MA Gimbrone Jr, Boston, MA, USA), mouse anti-human CD31 (M0823, Dako) to detect CD31 as a standard marker for endothelial cells, and mouse anti-rat ICAM-1 (1A29, kindly provided from Dr M Miyasaka, Osaka Univ., Osaka, Japan) as an iso-type control. After washing, cells were incubated for one hour with 100 μl rat anti-mouse F(ab')_2_-FITC (F0313, Dako). After extensive washing, cells were fixed with 0.5% formalin in PBS. Flow cytometric analysis was performed within 24 hours after fixation using a Coulter Epics-Elite flow cytometer (Coulter Electronics, Hialeah, FL, USA). Data were analyzed using Winlist (version 3D; verity Software House, Topsham, ME, USA) and WinMDI (version 2.8; The Scripps Research Institute, La Jolla, CA, USA) software.

### Statistical analysis

Statistical significance of differences was evaluated by means of the two-sided Student's *t *test, assuming equal variances. Differences were considered to be significant when *p *< 0.05.

## Results

### Effectiveness of the virally delivered dnIκB protein

**Figure 1:**
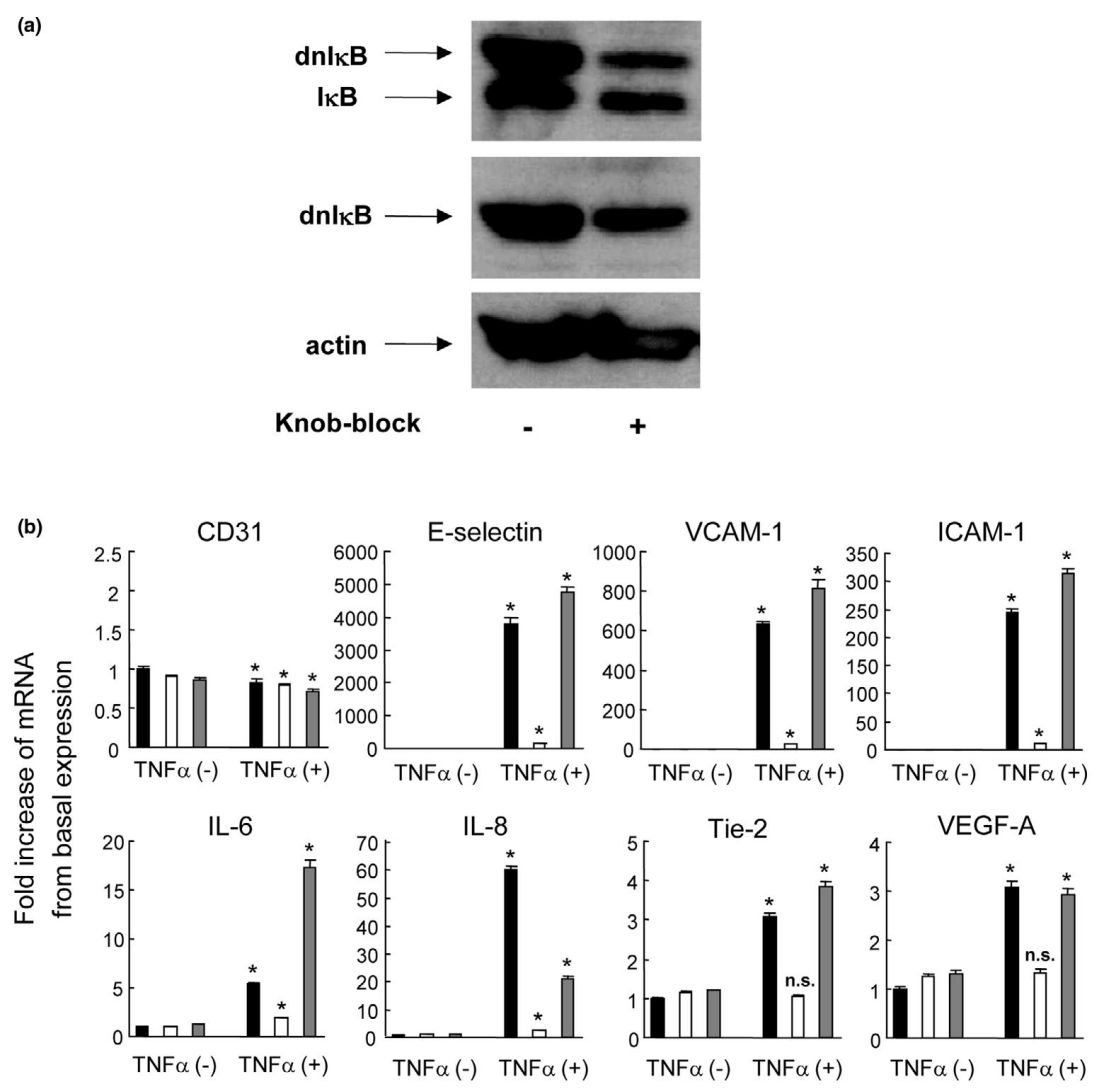
Human umbilical vein endothelial cells (HUVECs) express functional dnIκB protein upon AddnIκB infection as demonstrated by western blot analysis and gene expression analysis by real time RT-PCR. **(a) **HUVECs were incubated with AddnIκB for 90 minutes at 37°C, in the absence or presence of 20 mg/ml recombinant viral knob, as described in Materials and methods. Cells were subsequently washed and incubated for another 24 hours. After preparation of cellular protein homogenate, western blotting was performed to detect IκB total protein (upper panel), the hemagglutinin-tagged transgene dnIκB (middle panel), and actin to control for protein loading (lower panel). **(b) **Non-infected (solid bar) and AddnIκB (open bar) or AdLacZ (gray bar) transduced HUVECs were activated with tumor necrosis factor (TNF)-α (100 ng/ml) for 24 h before real time RT-PCR was performed on mRNA isolated from each respective HUVEC incubation. Data were normalized to untreated, non-activated control HUVECs arbitrarily set at 1. Results are expressed as the mean ± standard deviation (n = 3). Asterisks indicate *p *< 0.05 compared with respective control cells without activation with TNF-α (TNF-α (-)). ICAM, intercellular adhesion molecule; ns, not significant; VCAM, vascular cell adhesion molecule; VEGF, vascular endothelial growth factor.

For the functional validation of the virus itself, we first infected HUVECs with AddnIκB. Western blotting experiments showed that the transgene was successfully expressed upon infection. Furthermore, pre-incubation with recombinant knob strongly inhibited the transduction of AddnIκB while not affecting the expression level of endogenous IκB (Figure 1a). Neither non-infected nor AdLacZ-infected HUVECs expressed the transgene (data not shown). Several genes that are characteristic for the inflammatory responses in endothelial cells contain functional NF-κB binding sites in their promoter regions, leading to enhanced transcription upon NF-κB activation [13]. We therefore determined the pharmacological effects of dnIκB transgene expression in HUVECs by analysis of mRNA levels of typical cell adhesion molecules, cytokines and some other angiogenesis-related genes in HUVECs upon TNF-α stimulation (Figure 1b). TNF-α stimulation enhanced mRNA levels of all genes investigated in untreated HUVECs, ranging from 3,778-fold for E-selectin to 3.1-fold for VEGF-A, except for the mRNA level of CD31, the expression of which was non-responsive to TNF-α stimulation (Table 1). This transcriptional induction was completely abolished in dnIκB expressing HUVECs activated with TNF-α. In contrast, transduction with the control virus AdLacZ did not affect the TNF-α induced up-regulation of cell adhesion molecules and the angiogenesis-related genes encoding VEGF-A and Tie-2. In AdLacZ infected HUVECs, IL-6 and IL-8 mRNA levels exhibited higher and lower increases, respectively, upon TNF-α stimulation compared to uninfected HUVECs, which may be a result of viral infection *per se*. Yet, TNF-α driven increases in mRNA levels of these genes was completely abolished in dnIκB expressing HUVECs. The effect of AddnIκB or AdLacZ infection *per se *on basal mRNA expression in the absence of TNF-α was within 20% for all genes investigated. This implies that viral infection does not influence basal expression under the conditions studied and, furthermore, that the observed non-responsiveness of dnIκB expressing HUVECs to an inflammatory stimulus was due to NF-κB blockade, and not due to viral infection itself. In all conditions, >80% of the dnIκB expressing HUVECs remained viable, as assessed microscopically as well as by 3-(4,5-dimethylthiazol-2-yl)-2,5-diphenyl tetrazolium bromide (MTT) viability assay (data not shown).

**Figure 2:**
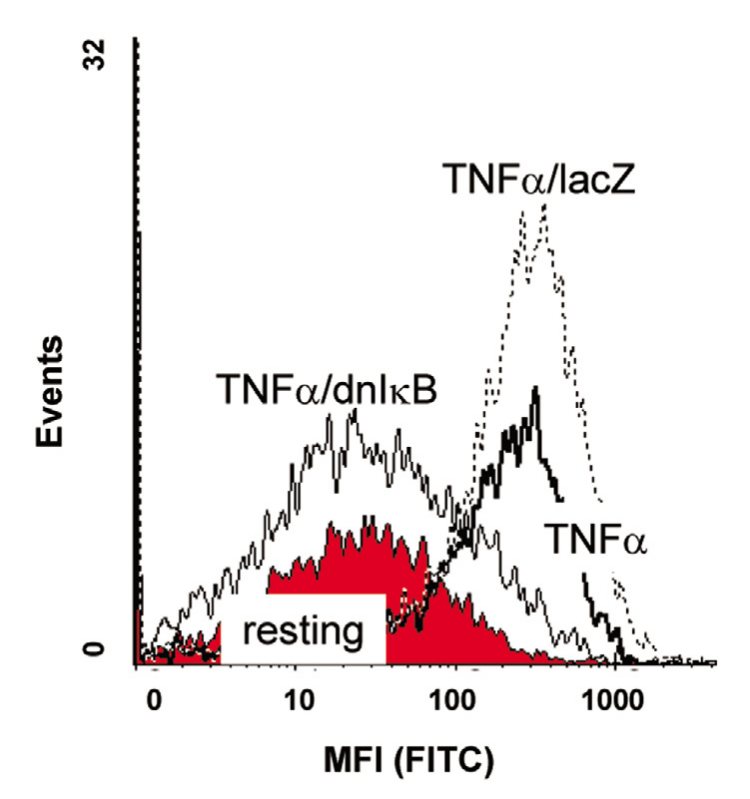
DnIκB expression in human umbilical vein endothelial cells (HUVECs) affects cellular responsiveness to tumor necrosis factor (TNF)-α activation, leading to diminished expression of intercellular adhesion molecule-1 protein as determined by flow cytometry. HUVECs were infected with AddnIκB (7,500 vp/cell) or AdLacZ (7,500 vp/cell). After 24 hours of culturing, cells were activated with 100 ng/ml TNF-α, or left resting. Cells were detached 4 hours after activation and subjected to flow cytometric analysis. Non-activated, resting HUVECs (solid line with gray area); TNF-α activated HUVECs (bold solid line); TNF-α activated HUVECs infected with AddnIκB (solid line); and TNF-α activated HUVECs infected with AdLacZ control virus (dotted line). FITC, fluoroscein isothiocyanate. MIF, mean fluorescence intensity.

To further confirm the inhibitory effects of the transgene on expression levels of NF-κB driven proteins, we determined the expression level of the transmembrane protein ICAM-1 (Figure 2), mRNA levels of which were shown to be silenced by the transgene (Figure 1b). TNF-α stimulation markedly induced the expression of ICAM-1 protein on the membrane of the endothelial cells. This expression was completely inhibited in HUVECs infected with AddnIκB prior to TNF-α stimulation, while no inhibitory effect was observed after pre-infection with control virus (AdLacZ). In contrast, the constitutively expressed endothelial gene CD31 was not affected by dnIκB (data not shown), thereby corroborating other observations that CD31 expression is NF-κB independent (JM Kuldo and G Molema, unpublished data). From these data, we concluded that the transgene employed could be functionally expressed in the primary endothelial cells without compromising cell viability.

### RGD-PEG modification endows adenovirus with αv integrin specific infectivity and transgene expression

**Figure 3:**
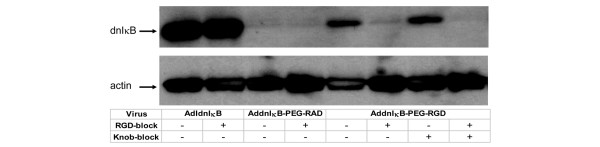
AddnIκB-PEG-RGD infected human umbilical vein endothelial cells (HUVECs) express dnIκB in a knob-independent, RGD-dependent manner. HUVECs were incubated with either non-modified AddnIκB, AddnIκB-PEG-RGD or AddnIκB-PEG-RAD (3,000 vp/cell) for 90 minutes, in the absence or presence of either 20 mg/ml recombinant viral knob or 50 mg/ml RGD-protein or both, as described in Materials and methods. Cells were subsequently washed and incubated for another 24 h. After preparation of cellular protein homogenate, western blotting was performed to detect the hemagglutinin-tagged transgene, and actin to control for protein loading.

We next confirmed the change in the entry pathway of RGD-retargeted adenovirus into HUVECs from a CAR-dependent to an αvβ3 integrin-dependent mode. Western blotting analysis of HA-tagged dnIκB (Figure 3) demonstrated that non-modified AddnIκB exhibited efficient transduction upon infection to HUVECs. The presence of exogenously added RGD-protein did not affect this transduction, suggesting that the entry pathway of non-modified virus is exclusively CAR-dependent. On the other hand, the transduction of HUVECs by AddnIκB-PEG-RGD was significantly inhibited by the presence of RGD-protein but not by recombinant knob5, while AddnIkB-PEG-RAD showed no transduction at all. These results strongly suggest that RGD modification successfully endowed adenovirus with αv integrin specific infectivity to endothelial cells, and that peptide modification *per se *was not responsible for directing the tropism of the virus.

### RGD-PEG modified adenovirus can transfer a functionally active dnIκB gene into endothelial cells

To study whether chemically modified AddnIκB exerted therapeutic potential for interference of inflammatory and angiogenic processes, we evaluated mRNA levels of the same set of genes investigated to study functionality of the non-modified virus.

**Figure 4:**
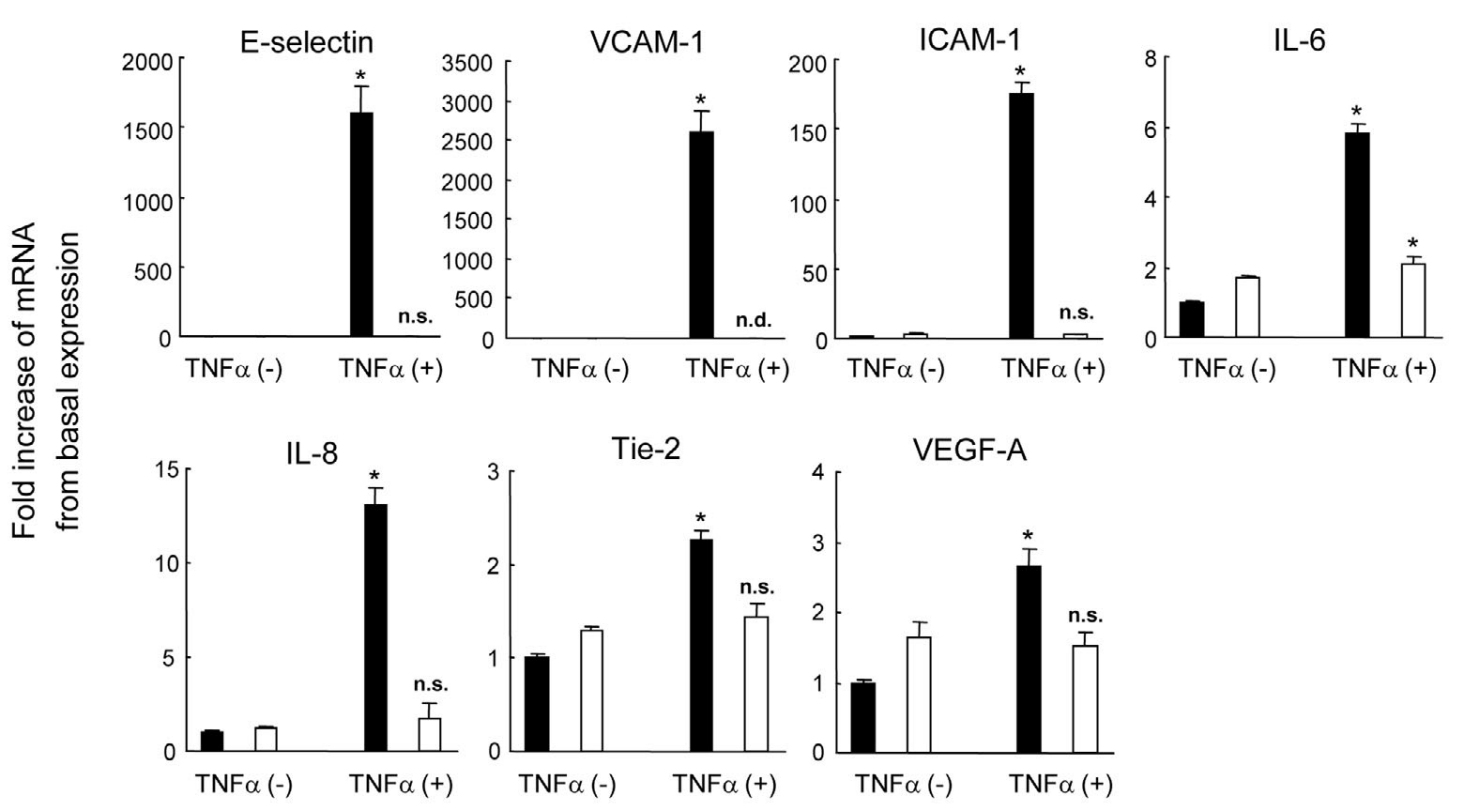
Inhibitory effects of αvβ3-retargeted adenovirus on tumor necrosis factor (TNF)-α induced gene expression of cell adhesion molecules, cytokines, and angiogenesis associated molecules in human umbilical vein endothelial cells (HUVECs). Non-transduced (solid bar) and AddnIκB-PEG-RGD transduced HUVECs (open bar) with 7,500 vp/cell were activated with TNF-α (100 ng/ml) for 24 h. Real time RT-PCR was performed on mRNA isolated from each respective HUVEC incubation. Data were normalized to untreated, non-activated control HUVECs arbitrarily set at 1. Results are expressed as the mean ± standard deviation (n = 3). Asterisks indicate *p *< 0.05 compared with respective control cells without activation with TNF-α (TNF-α (-)). ICAM, intercellular adhesion molecule; nd, not detectable; ns, not significant; VCAM, vascular cell adhesion molecule; VEGF, vascular endothelial growth factor.

Figure 4 shows that TNF-α driven expression of all pro-inflammatory and pro-angiogenic genes was completely abolished in HUVECs infected with the RGD-PEG modified virus. Moreover, AddnIκB-PEG-RGD exhibited a similar inhibitory effect on gene expression as the non-modified virus (Figure 1b).

**Figure 5:**
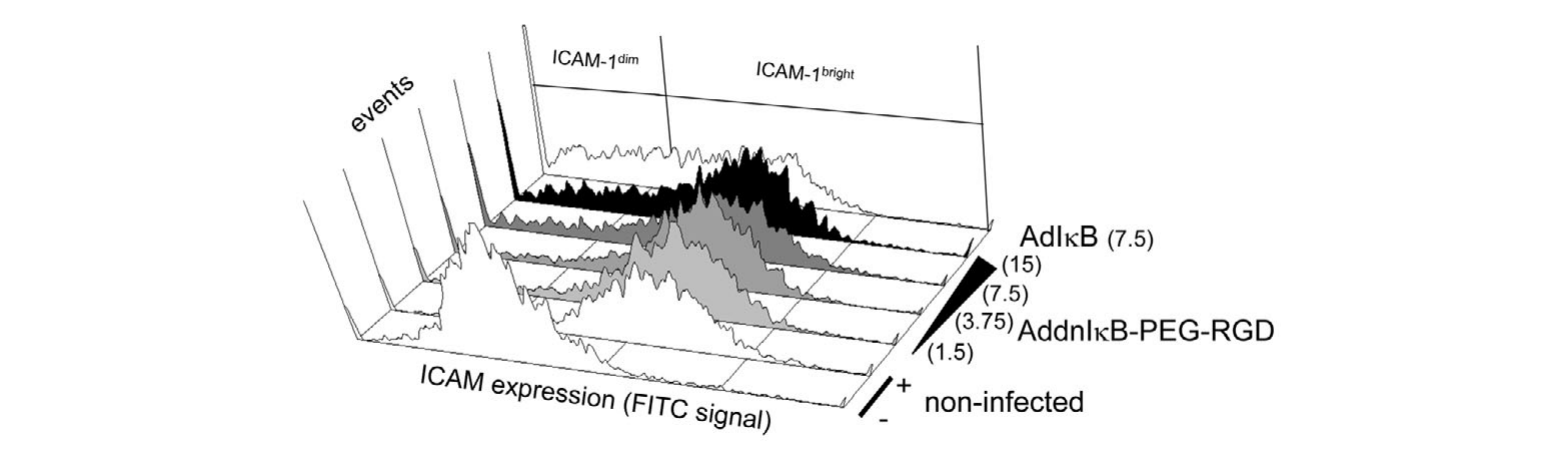
AddnIκB-PEG-RGD can transduce functional dnIκB in a concentration dependent way leading to diminished intercellular adhesion molecule (ICAM)-1 protein expression upon tumor necrosis factor (TNF)-α stimulation in human umbilical vein endothelial cells (HUVECs) as determined by flow cytometry. Minus and plus signs and denotes resting, non-infected, and TNF-α activated, non-infected HUVECs, respectively. All other histograms represent the responses of dnIκB expressing HUVECs to TNF-α activation. The larger the number of viral particles/cell (× 10^3^) of AddnIκB-PEG-RGD used for the infection, the higher the percentage of ICAM-1dim cells upon TNF-α activation.

mRNA data demonstrating that the chemically modified RGD-PEG-adenovirus could transfer functionally active dnIκB gene into endothelial cells were confirmed by the analysis of ICAM-1 protein expression (Figure 5). The larger the number of viral particles of AddnIκB-PEG-RGD used for the infection, the higher the percentage of ICAM-1dim cells (cells that do not express significant levels of ICAM-1 protein) upon TNF-α activation, ranging from 6% for HUVECs transduced at 1.5 × 10^3 ^vp/cell to 27% for HUVECs transduced at the highest number of viral particles, 15 × 10^3 ^vp/cell. AddnIκB-PEG-RAD did not show any significant inhibitory effect on ICAM-1 protein expression upon TNF-α stimulation (data not shown), which is in line with the absence of dnIκB protein expression in cells exposed to this control virus (see western blot analysis shown in Figure 3).

## Discussion

NF-κB is a transcription factor that controls the expression of cytokines, chemokines and endothelial cell adhesion molecules to facilitate leukocyte movement from the blood stream into the underlying tissue [18,19]. NF-κB controls the vicious circle of endothelial cell activation and leukocyte recruitment during chronic inflammation that can lead to hypoxic conditions, a prelude to the initiation of angiogenesis [4]. In the current study, we show that adenoviral vectors encoding dnIκB protein modified to selectively infect pro-angiogenic, αvβ3 integrin expressing endothelial cells can be therapeutically exploited to inhibit TNF-α induced NF-κB activation. As a result, mRNA levels of E-selectin, VCAM-1, ICAM-1, IL-6 and IL-8 were reduced to basal.

In contrast to the well-acknowledged role of NF-κB in inflammation [5,9-13,20], its involvement in angiogenesis has been studied in much less detail [21,22]. Several lines of evidence suggest a functional role for this transcription factor in capillary tube formation [23] and retinal neovascularization [24]. Cytokines are crucial participants in receptor-mediated intracellular signaling during the (patho)physiological processes in inflammation-associated cellular events. They affect the endothelial cells *per se *by inducing the expression of a complex array of genes, thereby changing the endothelial activation status and the balance between cell growth and differentiation and cell survival and cell death [25]. Among them, IL-6 and IL-8 are mainly produced by endothelial cells and are critical players in the initiation phases of immunity and inflammation. Besides its active role in inflammation, it has recently been recognized that IL-8 also has potent pro-angiogenic effects through the induction of endothelial cell proliferation and capillary tube organization [26]. Thus, inhibition of IL-8 expression is likely to have anti-angiogenic as well as anti-inflammatory effects. The strong inhibitory effects of dnIκB expression in endothelial cells on VEGF-A and Tie-2 gene expression further point to the potential consequences of this therapeutic strategy for inflammation induced angiogenesis. Vascular smooth muscle cells can also be the source of VEGF-A and, as such, can contribute to inflammation-induced angiogenesis. Angiogenesis often takes place in microvascular bed endothelial cells, however, where only sparsely distributed pericytes are covering the vessel wall in these capillaries [27]. Whether inhibition of microvascular, endothelial expression of angiogenic genes *per se *will suffice in counteracting the pro-angiogenic status of the tissue will be the focus of future *in vivo *pharmacological studies. An important advantage of the use of PEGylated virus is that PEGylated virus shows a significantly increased blood residence time in mice. The area under the plasma concentration time curve value was shown to be 17-fold increased compared to that of non-modified virus [15]. Extensive circulation ensures prolonged exposure of the target endothelial cells in the inflamed joint to the therapeutic gene vector, which may positively affect the therapeutic efficacy.

For selectivity of targeting, the discrimination between endothelial cells in chronic inflammatory, angiogenic lesions and the normal quiescent vascular endothelium is critical. In the past years, several target epitopes over-expressed on activated (for example, angiogenic or pro-inflammatory) endothelial cells have been identified, including αvβ3 integrins [28], E-selectin [29] and VCAM-1 [30]. We previously reported that anti-E-selectin antibody-directed PEGylated adenovirus selectively homed to inflamed skin in mice with a delayed type hypersensitivity skin inflammation. As a result, selective local expression of the reporter transgene luciferase took place [15]. Although E-selectin is present on endothelial cells in inflamed joints in mice suffering from arthritis, the number of capillaries positive for this potential target was found to be low [31]. As is the case in tumor vasculature, heterogeneity in endothelial activation status may also present itself during chronic phases of inflammation. Therefore, a multi-target approach should be considered to obtain optimal pharmacological effects.

In the present study, we demonstrated that chemically modified AddnIκB-PEG-RGD exhibited a shift in specificity of cell entry from its intrinsic CAR-driven entry pathway to an αv integrin-mediated pathway. Although our present study only dealt with HUVECs, our previous study showed that the RGD-PEG-adenovirus enabled transduction of the reporter gene luciferase in CAR-negative but αvβ3 integrin-positive mouse endothelial cells. Together with the observation that in the same CAR-negative cells no luciferase activity could be transduced by non-modified adenovirus, this implies that the transduction by the chemically modified virus is αvβ3 integrin specific [15]. This specificity furthermore means that *in vivo*, αvβ3 integrin-positive cells, including angiogenic endothelial cells, macrophages in spleen and liver, and macrophage subsets in the intestines [32] and also fibroblasts and macrophages that constitute the synovial lining [33,34], are likely to be the target for the modified virus. Our data also demonstrated that our chemically modified AddnIκB-PEG-RGD can exert pharmacological effects similar to those observed with the non-modified virus. An interesting observation was the fact that the amount of transgene protein required to inhibit NF-κB dependent gene transcription was much less than the amount of endogenous IκB present in the cells. Moreover, no linear relationship between the amount of dnIκB expressed in HUVECs (Figure 3) and the effect was observed (Figures 1b and 4). A similar anomaly between the degree of inhibition of IκB degradation and its effect on mRNA or protein expression level for several inflammation-related markers was previously reported by Liu and colleagues [35]. To investigate whether the absolute amount of dnIκB to be delivered *in vivo *will be sufficient to inhibit the inflammatory and/or angiogenic behavior of the endothelial target cells is an important issue to address and is the focus of future studies.

We focused our research on the delivery of therapeutic genes into endothelial cells, yet there is now considerable evidence in support of a role for NF-κB in synoviocyte survival as well [36]. By combining the therapeutic approach presented here with homing devices to, for example, target synoviocytes in the joint [37] or cells in the neointima in artery injury [38], a range of possibilities can be defined to explore the therapeutic benefit of targeted interference with different cells actively involved in joint destruction [39]. Since NF-κB has the dual function of being responsible for both tissue protection and systemic inflammation [40], targeted inhibition of NF-κB is vital to modulate the activation status of cells involved in disease progression while avoiding the detrimental effects of NF-κB blockade in non-target cells.

## Conclusion

RGD modification endowed PEGylated adenovirus with the specificity of cell entry via αvβ3 integrin, thereby avoiding its intrinsic coxsacki-adenovirus receptor controlled entry. RGD-targeted adenovirus delivered the dnIκB via αvβ3 to become functionally expressed leading to complete abolishment of TNF-α-induced up-regulation of E-selectin, ICAM-1, VCAM-1, IL-6, IL-8, VEGF-A and Tie-2 in HUVECs. The approach of targeted delivery of dnIκB into endothelial cells presented here can be employed for diseases such as rheumatoid arthritis and inflammatory bowel disease where activation of NF-κB activity should be locally restored to basal levels in the endothelium.

## Abbreviations

CAR = coxsacki-adenovirus receptor; Ct = threshold cycle; dn, dominant negative; FCS = fetal calf serum; HA = hemagglutinin; HUVEC = Human umbilical vein endothelial cell; ICAM = intercellular adhesion molecule; IL = interleukin; NF-κB = nuclear factor κB; PBS = phosphate-buffered saline; PEG = polyethylene glycol; RADpep = cyclic RAD peptide c(RADf(∊*-S*-acetylthioacetyl)K); RGDpep = cyclic RGD peptide c(RGDf(∊*-S*-acetylthioacetyl)K); RT-PCR = reverse transcription polymerase chain reaction; TNF = tumor necrosis factor; VCAM = vascular cell adhesion molecule; VEGF = vascular endothelial growth factor; vp = viral particles.

## Competing interests

The authors declare that they have no competing interests.

## Authors' contributions

K-iO and GM conceived the study, participated in its design and interpretation of data, and drafted the manuscript. K-iO and KO performed all the experiments. JMK executed the real time RT-PCR analysis. KO, JMK, BJK, MGR, CT, TK and HJH participated in different parts of the study, interpretation of data, and drafting the manuscript. All authors read and approved the final manuscript.

## Figures and Tables

**Table 1:** mRNA levels of the genes studied upon tumor necrosis factor-α activation

Gene product	Fold increase
CD31	0.8 ± 0.1
E-selectin	3,778 ± 200
Vascular cell adhesion molecule-1	637 ± 12
Intercellular adhesion molecule -1	245 ± 7.0
IL-6	5.3 ± 0.2
IL-8	9.9 ± 0.5
Tie-2	3.6 ± 0.1
Vascular endothelial growth factor-A	3.1 ± 0.1

Data are expressed as basal gene expression levels in non-stimulated human umbilical vein endothelial cells set at 1. Results are expressed as the mean ± standard deviation (n = 3).
